# Hospitalization for heart disease, stroke, and diabetes mellitus among Indian-born persons: a small area analysis

**DOI:** 10.1186/1471-2261-4-19

**Published:** 2004-10-27

**Authors:** Peter Muennig, Haomiao Jia, Kamran Khan

**Affiliations:** 1 Department of Health Policy and Management, Mailman School of Public Health, Columbia University, New York, NY 10003, USA; 2 Department of Community Medicine, Mercer University School of Medicine, Macon, GA, 31207, USA; 3 Inner City Health Research Unit, St. Michael's Hospital and the University of Toronto, Toronto, ON, M5V2M4, Canada

## Abstract

**Background:**

We set out to describe the risk of hospitalization from heart disease, stroke, and diabetes among persons born in India, all foreign-born persons, and U.S.-born persons residing in New York City.

**Methods:**

We examined billing records of 1,083,817 persons hospitalized in New York City during the year 2000. The zip code of each patient's residence was linked to corresponding data from the 2000 U.S. Census to obtain covariates not present in the billing records. Using logistic models, we evaluated the risk of hospitalization for heart disease, stroke and diabetes by country of origin.

**Results:**

After controlling for covariates, Indian-born persons are at similar risk of hospitalization for heart disease (RR = 1.02, 95% confidence interval 1.02, 1.03), stroke (RR = 1.00, 95% confidence interval, 0.99, 1.01), and diabetes mellitus (RR = 0.96 95% confidence interval 0.94, 0.97) as native-born persons. However, Indian-born persons are more likely to be hospitalized for these diseases than other foreign-born persons. For instance, the risk of hospitalization for heart disease among foreign-born persons is 0.70 (95% confidence interval 0.67, 0.72) and the risk of hospitalization for diabetes is 0.39 (95% confidence interval 0.37, 0.42) relative to native-born persons.

**Conclusions:**

South Asians have considerably lower rates of hospitalization in New York than reported in countries with national health systems. Access may play a role. Clinicians working in immigrant settings should nonetheless maintain a higher vigilance for these conditions among Indian-born persons than among other foreign-born populations.

## Background

Immigrant populations in industrialized nations are on average healthier than their native-born counterparts [1]. However, there is considerable debate surrounding the risk of ischemic heart disease and other related vascular and metabolic conditions among South Asian Indians, both within India and in the countries to which they immigrate [2-7]. The relationship between ischemic heart disease and Indian ethnicity is complex. For instance, lower rates of smoking are juxtaposed against higher mean cholesterol levels and unfavourable cholesterol ratios, higher rates of central obesity, and a higher prevalence of diabetes mellitus among Indian immigrants relative to either native-born or other foreign-born populations[3-5], [7-9].

Studies conducted in countries with national health-care systems have noted approximately twice the risk of hospitalization for ischemic heart disease or myocardial infarction among South Asian immigrant men relative to native-born men aged 45–64 [10]. This amounts to nearly four times the risk of Caribbean-born men in the same study. However, India-born women were found to be at similar risk as native-born women. South Asian immigrants may also experience the onset of symptomatic heart disease at a younger age than native born populations [11]. Nonetheless, mortality rates of ischemic heart disease among Indian immigrants to Canada were comparable to Canadians of European ancestry [12]. There is therefore considerable confusion surrounding the actual underlying risk of ischemic heart disease among South Asian persons.

We set out to describe relative differences in the rates of hospitalization for heart disease, stroke, and diabetes among Indian-born persons relative to native-born persons or other foreign-born persons living in New York City. Unfortunately, there are no national datasets that provide individual level data for the risk of hospitalisation among specific immigrant sub-groups. It is, however, possible to compare the risk of hospitalization among immigrant sub-groups on an area-level using established small area analytic techniques via local datasets with a large sample size [13]. Herein, we quantify the risk of hospitalization for heart disease among Indian-born persons relative to other groups using a large hospitalization dataset covering New York City.

## Methods

We obtained all hospital admission records for residents of New York City from the Statewide Planning and Research Cooperative System (SPARCS) and population data for New York City from Census 2000 [14]. The SPARCS dataset contains information on 2.45 million hospitalizations for the civilian population of New York State, including basic demographic variables and diagnosis codes [15]. A logistic model was used to calculate rate ratios and risk ratios (RR) rather than odds ratios. All analyses were conducted on SAS version 8 (Cary, NC).

We used this model to calculate hospitalizations for manifestations of atherosclerotic heart disease (myocardial infarction, angina, and congestive heart failure, International Classification for Disease, 9^th ^Revision codes 410, 413, and 428 respectively), stroke (430–438), and diabetes (250) as the dependent variables in each regression model. The following categorical variables were entered as independent predictors: age (0–6, 7–17, 18–44 [reference], 45–64, 65+), sex (reference = female), education level (completed high school versus no high school [reference]), and country of birth (born in India, other foreign-born persons, and native-born [reference]).

While the SPARCS dataset includes information on patients' age, sex, race, and ethnicity, it does not contain information on country of origin, income, or education level. To obtain country of origin and income data, we matched each patient's zip code from SPARCS hospitalization records to information obtained from the Census 2000 Long Form [14].

For instance, to calculate age-specific hospitalization rates, all hospitalizations among persons aged 65 and older residing within a particular zip code were divided by the number of persons aged 65 and older living within that same zip code. We then entered the proportion of foreign-born persons and Indian-born persons living within each zip code in New York City, as well as the proportion graduating from high school as independent variables in our regression analysis.

The model took the general form:



Logistic models were constructed for each category of hospitalization and the variables representing the proportion of persons from each region of the world. We tested the models using Hosmer and Lemeshow goodness of fit test, and index plots of the residuals.

## Results

In 2000, there were 70,598 persons in New York City who were born in India (see Table 1). Of these, 45% were female, 63% were married, and 14.4% were living below the poverty line. Table 2 presents the relationship between age, sex, and education level on the risk of hospitalization for heart disease alone. As expected, there is an increase in heart disease risk with increasing age, male sex, and lower educational attainment.

**Table 1:** Socio-demographic characteristics of Indian-born persons in New York City.

	**Number**	**Percent**
Total Population	70,598	-
Age		
0–17 years	7,489	10.6%
18–24 years	7,681	10.9%
25–34 years	17,950	25.4%
35–44 years	15,938	22.6%
45–54 years	11,789	16.7%
55–64 years	6,499	9.2%
65–74 years	2,320	3.3%
75 + years	932	1.3%
Sex		
Female	31,749	45.0%
Male	38,849	55.0%
Marital Status		
Now married	44,665	63.3%
Widowed	2,093	3.0%
Divorced	1,352	1.9%
Separated	561	0.8%
Never married*	21,927	31.1%
Poverty status		
Group quarters	583	0.8%
Below poverty line	10,150	14.4%
100–200% poverty line	11,797	16.7%
>200% poverty line	48,068	68.1%

*Includes persons < age 15.

**Table 2:** Risk ratios of hospitalization for heart disease by age, sex, and education (reference levels and 95% confidence interval are indicated in brackets)

Variable	Female	Male	Total
Age			
0–6	0.218 (0.153, 0.310)	0.534 (0.361, 0.788)	0.299 (0.230, 0.388)
7–17	0.192 (0.130, 0.285)	0.336 (0.126, 0.892)	0.219 (0.152, 0.316)
18–44 (ref)	1.00	1.00	1.00
45–64	13.58 (12.99, 14.19)	15.42 (14.42, 16.48)	13.98 (13.47, 14.51)
65+	44.75 (42.84, 46.75)	77.37 (72.55, 82.50)	55.67 (53.71, 57.70)
Male Sex (Female)	N/A	1.62 (1.60, 1.65)	N/A
High School Grad. (Non-Graduate)*	0.943 (0.935, 0.951)	0.857 (0.850, 0.865)	0.901 (0.896, 0.906)

*Because some values were small, risk ratios for this category were rounded to 3 decimal places.

Table 3 presents the risk of hospitalization for heart disease, stroke, and diabetes mellitus by country of birth controlling for age, sex, education, and percentage of foreign-born persons in zip code. After controlling for covariates, foreign-born persons were less likely than native-born persons to be hospitalized for heart disease (RR = 0.70, 95% confidence interval 0.67, 0.72). As with native-born persons, foreign-born women (RR = 0.67, 95% confidence interval 0.63, 0.71) were at lower risk of hospitalization for heart disease than foreign-born men (RR = 0.74, 95% confidence interval 0.70, 0.78); however, the difference was much smaller than it is with native-born persons (RR = 1.62, 95% confidence interval 1.60, 1.65, see Table 1). Persons born in India were at similar risk of hospitalization as native-born persons for heart disease (RR = 1.02, 95% confidence interval 1.02, 1.03), but 47% more likely to be hospitalized for heart disease than other foreign-born persons (data not shown). Differences in the risk of hospitalization between India-born men and women were minimal.

**Table 3:** Risk ratios of hospitalization for heart disease, stroke, and diabetes among Indian persons after controlling for age, sex, education, and percentage of foreign-born persons in zip code (reference levels and 95% confidence interval are indicated in brackets)

Variable	Male	Female	Total
Heart Disease			
Foreign-born (Native-born)	0.74 (0.70, 0.78)	0.67 (0.63, 0.71)	0.70 (0.67, 0.72)
Born in India (Native-Born)	1.03 (1.02, 1.04)	1.01 (1.00, 1.02)	1.02 (1.02, 1.03)
Stroke			
Foreign-born (Native-born)	0.75 (0.68, 0.83)	0.80 (0.73, 0.87)	0.78 (0.73, 0.83)
Born in India (Native-Born)	1.00 (0.98, 1.02)	0.99 (0.98, 1.01)	1.00 (0.99, 1.01)
Diabetes Mellitus			
Foreign-born (Native-born)	0.37 (0.33, 0.40)	0.42 (0.38, 0.47)	0.39 (0.37, 0.42)
Born in India (Native-Born)	0.97 (0.95, 0.99)	0.95 (0.92, 0.97)	0.96 (0.94, 0.97)

The risk of hospitalization for stroke is similar among India-born persons and native-born persons, with a risk ratio of 1 (95% confidence interval 0.99, 1.01). Indian-born persons, however, are more likely than other foreign-born persons to be hospitalized for stroke; the average foreign-born person has a risk ratio of 0.75 (95% confidence interval 0.73, 0.83).

While the risk of hospitalization for diabetes similar among Indian-born persons and native-born persons (RR = 0.96, 95% confidence interval 0.94, 0.97), the likelihood of hospitalization for this condition is considerably higher on average than that of other foreign-born persons regardless of sex. Foreign-born persons overall are at 39% the risk of hospitalization for diabetes mellitus (95% confidence interval 0.37, 0.42).

## Discussion

While Indian immigrants to industrialized nations are at similar risk of hospitalization for heart disease, diabetes, and stroke as their native-born counterparts, they are at much greater risk of hospitalization for heart disease, stroke, and diabetes than are immigrants from other countries. Although earlier studies have found differences in the risk of heart disease among Indian-born persons to England and Canada by sex, we found that risks were similar among males and females[10,11].

In an earlier paper, a higher age-adjusted mortality rate due to ischemic heart disease and stroke was noted among foreign-born women relative to native-born women [16]. In that study, it was postulated that the higher observed mortality rates might be due to changing demographic trends among recent immigrants. Specifically, it was hypothesized that mortality differences may be attributable to a higher proportion of female immigrants from higher risk countries. This hypothesis does not seem correct in light of current findings, and may be attributable to random error in the census sample.

Our study was subject to a number of important limitations. While the hospitalization data contained insurance status, the census data did not. We were therefore unable to control for insurance status in our study.

Given that foreign-born persons in the U.S. approach three times the rate of lacking health insurance as native-born persons (33.4% versus 12.2% respectively)[17], it is possible that many persons are at relatively lower risk of hospitalization and relatively higher risk of death. Still, since heart disease is a serious and potentially fatal condition, it seems unlikely that many immigrants would forgo hospital-based care for fear of incurring expenses. Moreover, the abundance of public hospitals in New York City reduces the probability that seriously ill persons would avoid seeking hospital care for fear of incurring medical costs. Nonetheless, some Indian-born persons may avoid seeking care due to a lack of health insurance. To the extent that avoidance of care occurs, the risk of hospitalization among Indian-born persons is underestimated in our analysis.

A second limitation was our use of zip-code-only data for the proportion of foreign-born in a neighbourhood, which may be confounded by ecological factors. This was minimized somewhat by examining individual-level hospitalizations for all variables but education and country of birth. It is notable that our data on the foreign-born overall are constant with a growing body of literature showing lower morbidity and mortality among immigrants to the United States than native-born persons[1].

The risk differences we found between native-born persons and persons born in India were not large – for every 100 hospitalizations for heart disease among native-born persons, we would expect 2–3 additional hospitalizations among persons from India. However, the risk of heart disease, cancer and diabetes is significantly higher than those of other immigrant groups with a similarly low prevalence of smoking[1]. Previous studies of behavioural risk factors suggest that diet is the overwhelming risk factor in the India-born population – a finding consistent with our subjects' higher risk of hospitalization for diabetes mellitus relative to native-born persons and other foreign-born persons [5,10,11]. The presence of a single overriding risk factor simplifies public health efforts; it is easier to target diet and exercise regimens, for instance, than a broader range of health behaviours.

## Conclusions

Indian-born New Yorkers are at substantially higher risk of heart disease, stroke, and diabetes than other foreign-born persons. However, these risks are comparable to that of heart disease, stroke, and diabetes among native-born persons. It is also conceivable that access to health services plays a role in the risk of hospitalisation for heart disease or related conditions given that higher hospitalization rates have been observed in countries with universal health coverage. Prospective data are needed to refine our understanding of the risk factors associated with heart disease in persons from India. As with interpreting data with any epidemiological study, results should not be generalized to clinical practice without first considering the individual patient's socio-demographic risk profile. Nevertheless, clinicians working in facilities serving foreign-born populations may wish to maintain a higher degree of vigilance for heart disease and its risk factors among persons from India.

## Competing interests

The author(s) declare that they have no competing interests.

## Authors' contributions

PM planned the analysis, outlined the study, reviewed the data and regression analyses, and contributed to the development of the manuscript.

HJ compiled the data, designed the small area analyses, and conducted the regression analyses.

KK contributed to the planning of the analysis and the preparation of the manuscript.

## Pre-publication history

The pre-publication history for this paper can be accessed here:


